# New Macrocyclic Amines Showing Activity as HIV Entry Inhibitors Against Wild Type and Multi-Drug Resistant Viruses^†^

**DOI:** 10.3390/molecules14051927

**Published:** 2009-05-22

**Authors:** Stefano Rusconi, Mirko Lo Cicero, Ottavia Viganò, Francesca Sirianni, Elisabetta Bulgheroni, Stefania Ferramosca, Andrea Bencini, Antonio Bianchi, Lidia Ruiz, Cecilia Cabrera, Javier Martinez-Picado, Claudiu T. Supuran, Massimo Galli

**Affiliations:** 1 Dipartimento di Scienze Cliniche “Luigi Sacco”, Sezione di Malattie Infettive e Immunopatologia, Università degli Studi, Ospedale Luigi Sacco, via G.B. Grassi 74, 20157 Milano, Italy; 2 Università degli Studi di Firenze, Laboratorio di Chimica Bioinorganica, Rm. 188, Via della Lastruccia 3, 50019 Sesto Fiorentino (Firenze), Italy; E-mail: claudiu.supuran@unifi.it (C-T.S.); 3 IrsiCaixa Foundation, University Hospital Germans Trias i Pujol, Badalona, Spain; E-mail: jmpicado@irsicaixa.es (J.M-P.)

**Keywords:** HIV-1, co-receptors, CXCR4, macrocyclic polyamines

## Abstract

Considering as a lead molecule the chemokine CXCR4 receptor antagonist AMD-3100, which shows significant anti-HIV activity *in vitro* and *in vivo*, we investigated a series of structurally related macrocyclic polyamines incorporating *o,o’*-phenanthroline or 2,2’-bipyridyl scaffolds as potential antiviral agents with lower toxicity and increased activity against both wild type X4-tropic and dual tropic HIV strains. The antiviral activity of these compounds was evaluated by susceptibility assays in PBMC (Peripheral Blood Mononuclear Cells) and compared to that of AMD-3100. The newly investigated compounds showed IC_50_s values in the low micromolar range and significantly inhibited the viral replication of wild type X4-tropic isolate and dual tropic strains. These macrocyclic polyamines constitute a promising class of HIV entry inhibitors.

## 1. Introduction

The World Health Organization (WHO) has estimated that about 33 million people are currently infected with HIV (Human Immunodeficiency Virus), the causative agent of AIDS (Acquired ImmunoDeficiency Syndrome). This syndrome remains one of the worst health problems of our era [1]. Highly Active Anti-Retroviral Therapy (HAART) as well as the development of new drugs have decreased in a remarkable way the morbidity and mortality from the infection by HIV. Most of the compounds used in clinic and able to block the viral replication act as inhibitors of the three essential viral enzymes, including protease (PR), transcriptase (RT) and integrase (IN) [2]. So far, full treatment efficacy has not been achieved because of the emergence of drug-resistant viruses [3,4,5]. It has been reported that selection of resistance mutations due to drug pressure with HAART causes therapeutic failure in at least 40-50% of patients [6].

In the last few years, many efforts have been made to find new pharmacological approaches in blocking the infection. Inhibition of HIV-1 entry into target cells is one of the therapeutic objectives [7]. The complexity of this process is an obstacle to the development of new entry inhibitors, but lessons can be learned from endogenous molecules that interfere with the early steps of the viral life cycle. HIV-1 gets into target cells after sequential interactions of the viral envelope glycoprotein with CD4 and a co-receptor such as the chemokine receptors CCR5 or CXCR4 [8]. The discovery of these co-receptors provided clarity in understanding the mechanism of viral entry and viral evolution, rendering them novel interesting targets for therapeutics in HIV-1 infection [9]. Different strategies in blocking HIV entry were considered either by targeting one of the cellular receptors, CD4 or the above mentioned chemokine co-receptors, or the envelope proteins. The majority of available compounds interfere with the binding of gp120 with the co-receptor. The aim of our study was to investigate compounds which may interfere with some of the first phases of HIV-1 life cycle. 

**Figure 1:**
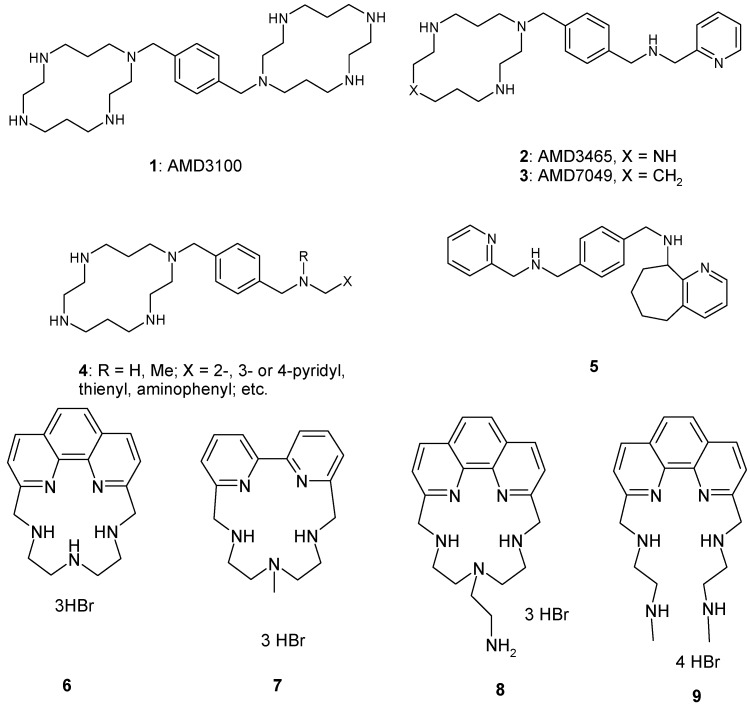
AMD3100 and related compounds plus the four newly synthesized macrocyclic polyamines.

Indeed, AMD3100 (**1**, Figure 1) was the first chemokine receptor antagonist to enter clinical studies for the treatment of AIDS/HIV infection [10] (the compound reached Phase II clinical studies, then further development was stopped due to its cardiotoxicity) [11]. AMD3100 is a bicyclam derivative possessing strong anti-HIV activity due to its inhibition of viral protein – CXCR4 interaction, with an IC_50_ of 2 – 20 nM (depending on the viral strain) [10,12,13,14,15,16,17]. This compound is active only against T-lymphocyte-tropic CXCR4-using viruses, and inactive against CCR5 or M-tropic viruses. AMD3100 has been recently approved for by the Food and Drug Administration for hematopoietic stem cell mobilization [18]. However, AMD3100 has been widely used as a lead compound for obtaining second generation CXCR4 antagonists, such as, among others, AMD3465 [19] (**2**) and several congeners **3-5** (Figure 1) possessing different heterocyclic/aromatic moieties in their molecules, which all show strong antiviral properties, with EC_50_ values in the range of 0.008 – 0.20 μg/mL and lower toxicity, compared to the lead compound [12,13,14,15,16,17]. Novel monomacrocyclic anti-HIV agents specifically block the interaction between HIV gp120 and CXCR4. Oral bioavailablility has not yet been achieved and this would be regarded a key feature to produce a better toxicity profile.

Considering AMD3100 (**1**) as lead compound, we investigate herein some polyamines (structures **6-9**, Figure 1) structurally related to this bicyclam as inhibitors of HIV-1 replication. In addition, this study aimed at considering the phenotypic susceptibility of HIV-1 isolates at different concentrations of such inhibitors, which may target the drug resistance problem which has emerged with many classes of antivirals [1,2,20,21,22,23].

## 2. Results and Discussion

### 2.1. Chemistry

Congeners of AMD3100 such as compounds **2-4** incorporate only one macrocyclic (14-membered) cyclam ring in their molecule, in contrast to the lead **1**, which incorporates two such rings. Furthermore, the last generation-such derivative, **5**, does not contain the macrocycle moiety in its molecule at all, but rather it is a tetra-amine derivative incorporating both aliphatic as well as aromatic (pyridyl) moieties [10,12,13,14,15,16,17]. Considering these derivatives as lead molecules we decided to investigate compounds which show some structural similarity with **1**-**5** as potential antiviral agents. Indeed, macrocyclic amines **6-9** investigated here, and reported earlier as metal complexing agents for supramolecular chemistry [24,25,26,27], contain a 15-membered macrocyclic ring based on o,o’-phenanthroline (in **6, 8 **and **9**) or a simplified 2,2’-bipyridyl scaffold (in **7**), rather similar to the bicyclam ring present in **1, 2 **and **3**. Furthermore, the secondary/tertiary amine functionalities thought to be important for the interaction of the drug with the CXCR4 antagonist [10,12,13,14,15,16,17] are also present in all the investigational compounds **6-8** used here. It should be also noted that in **9** the macrocyclic moiety is open, leading this to a compound with a less rigid structure, which is important to investigate as possible CXCR4 antagonist as compared to the cyclic congeners **6-8**. The other structural variations present in this small set of compounds regards one of the secondary amine endocyclic moieties, which is free amine in **6**, a methylamino group in **7** and an aminoethylamino moiety in **8** (obviously the open ring of **9** has a completely different structural scaffold in this part of the molecule).

**Scheme 1:**
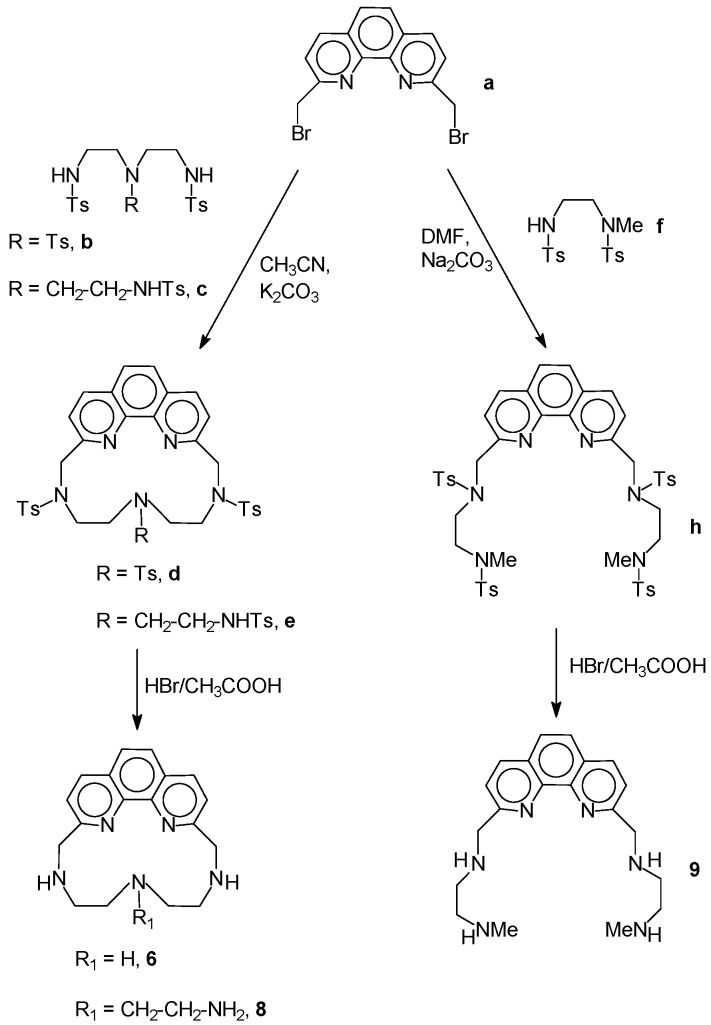
Synthetic procedures for compounds **8** and **9.**

Using AMD3100 (AnorMED, Langley, BC, Canada) as lead molecule, we investigated the structurally related macrocyclic polyamines **6-9**, reported earlier [24,25,26,27], in order to obtain compounds with lower toxicity or increased activity against both wild type isolates and viruses resistant to this class of drugs. Polyamines **6-9** are water soluble (as hydrobromides) making them interesting clinical candidates. The phenanthroline-based polyamines **6** [24], **8 **[27] and **9** [25] were synthesized by reaction of 2,9-bis(bromomethyl)-1,10-phenanthroline (**a**) [28] with 1,4,7-tritosyl-1,4,7-triaazaeptane (**b**) [29], tris[2-(*N-*tosylaminoethyl)]amine (**c**) [30] or 1-methyl-1,4-ditosyl-1,4-diazabutane [31] (**f**), respectively, in anhydrous CH_3_CN or DMF in the presence of Na_2_CO_3_ or K_2_CO_3_ as bases (Scheme 1). The resulting tosylated derivatives **d**, **e** and **h** were then deprotected in HBr/CH_3_COOH affording compounds **6**, **8** and **9**, respectively, as the corresponding trihydrobromide (**6**, **8**) or tetrahydrobromide (**9**) salts.

**Scheme 2:**
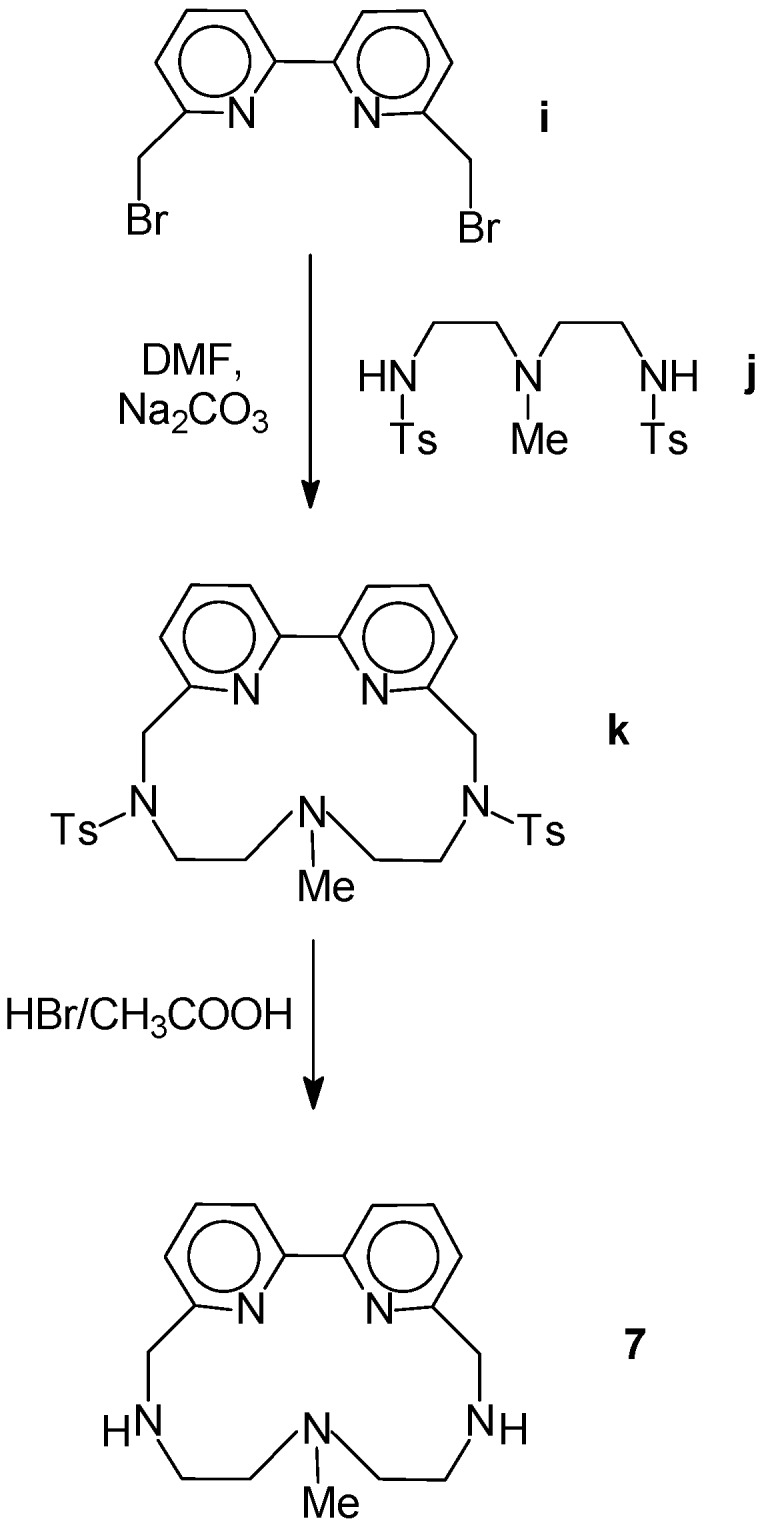
Synthetic procedure used for compound **7**.

The dipyridyl-containing macrocycle **7** [26] was obtained by reaction of 6,6’-bis(bromomethyl)-2,2’-bipyridyl [26] (**i**) with 4-methyl-1,4,7-tritosyl-1,4,7-triazaeptane [32] (**j**) in anhydrous DMF in the presence of Na_2_CO_3_ as base. The resulting tosylated precursor **k **was subsequently deprotected in HBr/CH_3_COOH to give polyamine **7** as the trihydrobromide salt (Scheme 2).

### 2.2. Definition of co-receptor usage

We defined dual tropism as the capacity of a certain HIV isolate to use CCR5 and CXCR4 as co-receptors. The large majority of our patients (six out of nine) were infected with a CCR5-tropic isolate, with only three patients (T01, T03, and RCP) showing a X4/R5 dual tropic virus. These three isolates showed a predominance of X4 usage (about 10-fold higher use of X4 than R5) without any change respect to W0. The prototype isolate 14aPre has been classified as X4-tropic.

### 2.3. Antiviral assay

#### 2.3.1. *In vitro* antiviral activity on a wild type HIV strain

The IC_50_s obtained for compounds **6-9** in infections mediated by a HIV-1 wild-type strain (14aPre) were in the low micromolar range, i.e.: 2.595 µM for molecule **6**, 2.436 µM for **7**, 3.511 µM for **8**, and 3.084 µM for **9**, as reported in Table 1. 

**Table 1:** 14aPre phenotypic susceptibility.

Compound	HIV-1 (14aPre)^a^	
	IC_50_(µM)^b^	SEM (µM)^c^
AMD3100	0.829	1.304
Compound #6	2.595	0.615
Compound #7	2.436	1.304
Compound #8	3.511	1.144
Compound #9	3.084	1.001

^a^ 14aPre: prototypic drug-sensitive isolate; ^b ^IC_50_: 50% inhibitory concentration, or concentration of compound required to inhibit 50% replication of virus, as determined by the susceptibility tests; ^c^ SEM: standard error of the mean.

No PBMC toxicity appeared at the highest drug concentrations (10 µM). In preliminary toxicity experiments, we used these compounds at concentrations up to 100 μM without any toxic effects in PBMC. AMD-3100 could inhibit the HIV-1 wild-type strain at nanomolar concentration (IC_50_ = 0.829 µM). The newly synthesized compounds resulted to be effective in inhibiting a prototypic HIV-1 isolate with a high sensitivity index. The highest mean inhibition was observed with compound **7** albeit without a statistically significant difference between compound **7** and the other investigated molecules. All the molecules tested were active in the micromolar range, even though AMD3100 showed an activity 2-3 times higher than the synthesized compounds.

#### 2.3.2. *In vitro* antiviral activity on dual tropic isolates

To estimate the efficacy of the four newly investigated polyamines in HIV-1-infected patients, their activity was examined against three dual tropic X4/R5 HIV-1 clinical isolates in PBMC from different healthy donors. The macrocyclic polyamines could inhibit the replication of all three X4/R5 isolates, with IC_50_s within a micromolar range. There was not a complete inhibition of viral replication (90% inhibition at the higher doses), since these dual tropic viruses are likely to replicate through the CCR5 receptor which the compounds do not inhibit. There was no difference in the antiviral activity of the new polyamines over time, *i.e.* week 0 and week 24 – 48, as indicated in Table 2. The IC_50_ ranges were 2.36 – 3.55 μM and 2.62 – 3.34 μM, respectively. On the contrary, the macrocyclic polyamines did not inhibit the replication of R5-tropic isolates in PBMC even at the highest concentration (10 µM) tested (data not shown). Thus, the 4 newly investigated macrocyclic polyamines **6-9** showed a promising inhibitory activity against dual-tropic HIV-1 isolates.

**Table 2:** Phenotypic susceptibility of the newly synthesized macrocyclic polyamines.

Patient^a^	IC_50_ values (week 0)^b^	IC_50_ values (week 24 - 48)^c^
TO1	2.43 - 3.51 µM	2.62 - 3.33 µM
T03	2.36 - 3.22 µM	2.72 - 3.34 µM
RCP	2.63 - 3.55 µM	2.72 - 3.34 µM

^a^ T01, T03, and RCP: dual tropic HIV-1 clinical isolates ; ^b,c^ Antiviral activity of the four polyamines on isolates from different time-points of HAART; expressed as IC_50 _ranges.

## 3. Experimental

### 3.1. Anti-HIV activity assays

#### 3.1.1. Viruses

Samples from baseline (prior to HAART treatment) up to week 96 were considered. The viruses derived from HIV-1-infected patients receiving HAART including T-20 and having detectable HIV-RNA. These isolates were either 3 class-experienced or harbored multi-drug resistance (MDR) HIV-1. Viruses of 8/9 patients showed an incomplete viral suppression at week 24 and were viremic at the end of the clinical observation; CD4 cell counts were generally well maintained with levels between 200 and 500/μL. The sequences (T01-T07, RCP, and XCA) have been submitted to GenBank (Bethesda, MD) (accession nos. from **DQ470847** to **DQ470872**). Moreover, we evaluated the isolate 14aPre, derived from an HIV-1 infected individual before any antiretroviral therapy (Massachusetts General Hospital, Boston, MA) and considered a drug-sensitive isolate.

#### 3.1.2. Co-receptor usage

All isolates were examined for their amino acid sequence and the co-receptor usage (CXCR4 and CCR5). The supernatant fluids from PBMC cultures infected with or without the inhibitors were used to infect U87MG-transformed CD4+ cells transfected with CCR5 or CXCR4 coreceptors [33]. We verified the viral co-receptor use as previously described [34], using p24 Ag as measurement endpoint.

#### 3.1.3. Compounds formulation

AMD-3100, a bicyclam compound active against X4-tropic viruses, was used at concentrations ranging from 0.025 µM to 0.2 µM and the four macrocyclic compounds from 2.5 to 10 µM. Each compound was tested as follows: AMD-3100 was ranged from 0.025 µM to 0.2 µM (0.025, 0.05, 0.1, 0.2 µM) and the four compounds from 2.5 to 10 µM (2.5, 5, 7.5, 10 µM). Compounds were diluted in DMSO at a concentration of 10 mM and stored at –20°C until use.

#### 3.1.4. Susceptibility assays

In each drug study, 3- or 4-day PHA-stimulated PBMC from donors were exposed to the HIV-1 inoculum (1,000 TCID50/ml per 10^6 cells) without a subsequent wash and a multiplicity of infection of 0.01 TCID50/cell [35]. Drugs were added simultaneously. Cells were suspended in a 1.0 mL final volume of R-20 medium supplemented with 10% interleukin-2 in 24-well tissue culture plates and incubated in a humidified atmosphere with 5% CO2 at 37°C. In all experiments culture medium was changed twice weekly so that 0.5 mL of cell suspension was resuspended in 1.0 ml of fresh medium that contained the original drug concentration(s). The drug concentrations that inhibited the viruses were evaluated in PBMC according to the method previously described [36,37]. Each single drug was tested in duplicate and each experiment was repeated at least twice. In addition, uninfected drug-treated toxicity controls were maintained at the highest concentration for each agent studied (either alone or in combination). We also maintained viruses without cells or drugs for the entire duration of the experiments in order to take into account the viral carryover.

## 4. Conclusions

Our cohort of subjects belonged to a large group of drug-experienced patients followed at our two Institutions. These patients had been heavily pre-treated with all ARV classes in the past. After checking the viral coreceptor usage of isolates derived from PBMC, we conducted *in vitro* susceptibility experiments in R5X4-tropic virus infections. The susceptibility experiments were conducted on four new derivatives containing different macrocyclic moieties based on 1,10-phenanthroline and 2,2’-bipyridyl scaffolds (some of which completely devoid of the macrocycle ring) as compared to the lead AMD3100. Such derivatives showed a very good water solubility (as hydrochloride/hydrobromide salts) and possess a lower molecular weight, which may be significant for their potential development as antivirals. The present study demonstrated the inhibition of isolates using the CXCR4 receptor by these macrocyclic polyamines in a wild-type prototypic isolate and in dual-tropic isolates derived from multi-drug experienced patients. These macrocyclic polyamines inhibited the replication of 14aPre in repeated experiments. The IC_50_s obtained for the four new compounds were in the low micromolar range (2.436 - 3.511 µM). It is also noteworthy that the isolates which these macrocyclic polyamines had been challenged against were multidrug-resistant strains, which were resistant to most of the traditional enzymatic HIV-1 inhibitors after *in vivo* virologic failure. These four macrocyclic polyamines inhibited three dual-tropic isolates with concentrations ranging from 2.36 to 3.55 µM and these values did not dramatically change after 24 to 48 weeks of unsuccessful treatment *in vivo* (range 2.62 – 3.34 µM).

Since the mode of action of these compounds is unrelated to those of the currently available anti-HIV-1 drugs, such as nucleoside or nucleotide reverse transcriptase (RT) inhibitors, non-nucleoside RT inhibitors, protease inhibitors, and the gp41-mediated fusion inhibitor T-20, it is conceivable that the combination between these new macrocyclic polyamines and the agents belonging to the other classes might lead to favourable synergistic results. Such examples came from our group [20], from work using SCH-C plus other antiretrovirals [21] and TAK-220 + T-20 [22], and TNX-355 and T-20 [23]. Taking the effective *in vitro* anti-HIV-1 properties into consideration, these macrocyclic polyamines represent a new promising class of HIV-1 entry inhibitors and warrant a deeper analysis. In perspective, we are conducting further drug combination experiments with wild-type and resistant HIV-1 isolates to better characterize these CXCR4 receptor antagonists. Also, we are using these results to synthesize other new compounds which hopefully will present even better performances, *i.e.* a greater antiviral activity and a more favorable toxicity profile.
